# Environmental distribution of certain modified live-virus vaccines with a high safety profile presents a low-risk, high-reward to control zoonotic diseases

**DOI:** 10.1038/s41598-019-42714-9

**Published:** 2019-05-01

**Authors:** Jennifer R. Head, Ad Vos, Jesse Blanton, Thomas Müller, Richard Chipman, Emily G. Pieracci, Julie Cleaton, Ryan Wallace

**Affiliations:** 1 Division of Global Health Protection, Center for Global Health, Centers for Disease Control and Prevention, Atlanta, GA USA; 2 Public Health Institute, San Francisco, CA USA; 3 IDT Biologika GmbH, 06861 Dessau, Rosslau Germany; 4 Division of High-Consequence Pathogens and Pathology, National Center for Emerging and Zoonotic Infectious Diseases, Centers for Disease Control and Prevention, Atlanta, GA USA; 5 Friedrich-Loeffler-Institut, Federal Research Institute for Animal Health, WHO Collaborating Centre for Rabies Surveillance and Research, Greifswald, Insel Riems Germany; 6 Wildlife Services Rabies Management, Animal Plant and Health Inspection Service, United States Department of Agriculture, Concord, NH USA

**Keywords:** Viral infection, Ecological modelling

## Abstract

Oral vaccines aid immunization of hard to reach animal populations but often contain live-attenuated viruses that pose risks of reversion to virulence or residual pathogenicity. Human risk assessment is crucial prior to vaccine field distribution but there is currently no standardized approach. We mapped exposure pathways by which distribution of oral vaccines may result in inoculation into people and applied a Markov chain to estimate the number of severe adverse events. We simulated three oral rabies vaccination (ORV) campaigns: (1) first generation ORV (SAD-B19) in foxes, (2) SAD-B19 in dogs, and (3) third generation ORV (SPBN GASGAS) in dogs. The risk of SAD-B19-associated human deaths was predicted to be low (0.18 per 10 million baits, 95% CI: 0.08, 0.36) when distributed to foxes, but, consistent with international concern, 19 times greater (3.35 per 10 million baits, 95% CI: 2.83, 3.98) when distributed to dogs. We simulated no deaths from SPBN GAS-GAS. Human deaths during dog campaigns were particularly sensitive to dog bite rate, and during wildlife campaigns to animal consumption rate and human contact rate with unconsumed baits. This model highlights the safety of third generation rabies vaccines and serves as a platform for standardized approaches to inform risk assessments.

## Introduction

While animal vaccination is an effective means of protecting human health through the reduction of zoonotic pathogens, vaccination of wildlife or free-roaming domestic animals (e.g. stray dogs) through subcutaneous or intramuscular injection remains a challenge. This is especially salient considering that 60–75% of emerging infectious diseases are caused by zoonotic pathogens^1^. Distribution, either aerially or as a hand-out, of oral animal baits containing live-virus vaccines has been used commercially and experimentally in both free-roaming dogs and wildlife populations against a variety of diseases, including rabies, brucellosis, Lyme disease, bovine tuberculosis, plague, and anthrax^2–6^.

However, due to the presence of live, replication-competent organisms in distributed vaccines, there is concern that their unsupervised presence in the environment may result in unintended severe adverse events (SAEs) in humans and animals (target species or not). Even in the highly touted examples of oral smallpox vaccination and oral polio vaccination, serious concerns about the safety of the live modified virus vaccines have arisen. For instance, widespread use of the vaccina virus against smallpox was originally associated with severe adverse events, prompting the development of second and then third generation vaccines to limit the number of persons who might experience adverse reactions^7^. In addition, current evidence shows that fecal shedding of the oral polio vaccine by vaccinated children has been associated with infection of unvaccinated children in the same household^8^.

Oral rabies vaccination (ORV) of wildlife and dogs is a prime example of the use of oral vaccine baits to control and eliminate the circulation of a deadly zoonosis within its animal reservoir. Distribution of oral rabies vaccines has effectively reduced the incidence of rabies in some terrestrial carnivores and has been credited with the near elimination of red fox-mediated rabies in Europe^9^. Several modified, live-attenuated, rabies vaccines have been developed for use, derivatives of the SAD (Street Alabama Dufferin) virus^10,11^, as well as newly generated live-recombinant vaccines^12,13^. For distribution, these vaccines are typically in liquid format, contained within a sachet or plastic blister pack, and incorporated in a bait. The World Health Organization (WHO) states that any oral rabies vaccine considered for field use should not induce any adverse health impacts in either target or non-target species, including people living within the area where baits are to be distributed^10^. Several attenuated SAD-derived first generation oral rabies vaccines show residual pathogenicity in adult mice^14–16^, and have been associated with rare vaccine-induced rabies cases in wildlife and domesticated animals^17–20^. Due to this potential residual pathogenicity, more attenuated strains and recombinant vaccines for use in wildlife populations have been developed^21^. Second generation vaccine strains were developed through selection of monoclonal antibody escape mutants targeting the amino acid position 333 of the rabies glycoprotein rendering the rabies virus apathogenic in immune-competent mice even after direct intracerebral inoculation^22–24^. Third generation vaccines, such as SPBN GASGAS, derived from the SAD complementary DNA (cDNA) clone^25^, introduce further alterations through reverse genetics to eliminate the risk of reversion to virulence (i.e. vaccine-induced rabies) and have been proposed for use in both wildlife and free-roaming dogs^26,27^. Laboratory studies suggest a lack of pathogenicity in healthy adult mice and find no evidence of inducing rabies in animals^2,26,28,29^. In 2017, SPBN GASGAS, was approved for commercial use in foxes and raccoon dogs (Nyctereutes procyonoides) in Europe^30^.

Proving the human safety of oral, baited vaccines is a crucial concern, and lack of robust risk assessment methods to do so can pose a significant barrier to the deployment of these vaccines^31^. While guidance for assessing the safety of oral vaccines is provided by the World Health Organization (WHO) and the World Organization for Animal Health (OIE), there is no standardized method for estimating the expected human health consequences nor is there a standardized method for comparing different oral rabies vaccines under differing settings and for differing target species. We developed a universal pathway describing comprehensive modes by which oral vaccines may cause a severe adverse reaction in humans and applied a continuous-time stochastic Markov chain model to explore the human safety of environmentally-distributed vaccines. We compared two commercially available oral rabies vaccines with a differing safety profile, SPBN GASGAS (Rabitec, IDT Biologika; Dessau-Rosslau, Germany) and SAD-B19 (Fuchsoral, IDT Biologika; Dessau-Rosslau, Germany), in two target animal populations (foxes and dogs) as a case study.

Using our exposure pathway model and Markov chain, we simulated the expected and 95% confidence intervals of the number of SAEs and the additional number of health care visits due to exposures to oral rabies vaccines during a 400,000 bait campaign. We then assessed safety differences in vaccine types and target populations by comparing model-simulated results from SAD-B19 in fox populations to SAD-B19 in free-roaming dog populations, and to SPBN GASGAS in free-roaming dog populations. The results of this analysis can be used by rabies program managers when considering vaccine options and weighing the risk of the vaccines against the risk posed by endemic rabies in the country. This approach could have application to other vaccine-preventable animal diseases.

## Methods

### Study Design/Model Development

**Figure 1:**
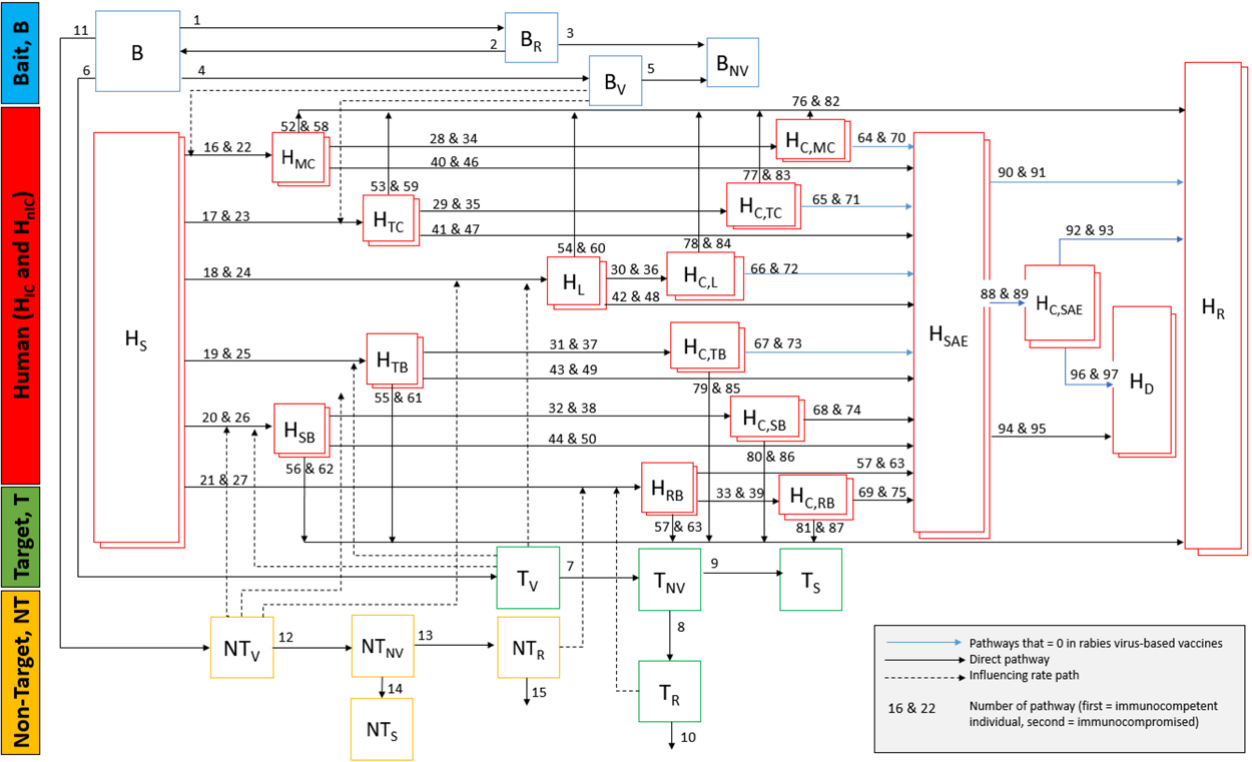
Compartmental model demonstrating route of oral vaccine in the environment and six potential routes of exposures.

The compartmental model in Fig. 1 describes movement of the vaccine-laden bait in the environment and the possibilities for human exposure to the vaccine due to direct transdermal or mucosal contact with a vaccine-laden bait; transdermal or mucosal contact with animals with residual vaccine in the oral cavity after bait consumption; or bite from an animal that experienced vaccine-induced rabies. Transdermal contact is considered that which penetrates the skin, either through puncture (as a dog bite), or via a skin lesion. Mucosal contact is considered contact with a mucosal membrane, such as the mouth, nose, or eyelids. Each box in Fig. 1 represents a state, or compartment (e.g. susceptible human, exposed human, vaccinated dog, consumed bait), containing a dynamic number of baits, humans, target animals or non-target animals. Compartment descriptions are outlined in Table 1. Solid arrows reflect movement of the person, animal, or bait out of one compartment and into another. Numbers above the arrows correspond to the rate of movement between each compartment, where mathematical equations for the rates are shown in Supplemental Table 1, and point estimates and uncertainty ranges for the parameters within these equations are shown in Supplemental Table 2. Blue arrows reflect rates that are zero for the SPBN GASGAS model. Dashed arrows originating from one compartment and leading to a solid rate arrow indicate that the dynamic value within the compartment of origin directly influences the rate at which movement between the two different compartments occur (e.g. the number of dogs that have consumed the bait is directly proportional to the number of humans that become exposed to the vaccine via dog bite).

**Table 1:** Definition of model compartments, shown in Fig. 1.

Variable	Description
B	Bait to be distributed
B_R_	Recovered bait
B_V_	Unrecovered, viable bait
B_NV_	Unrecovered, non-viable bait
H_S_	Unexposed and susceptible (nonvaccinated) immuno-competent/immuno-compromised human
T_V_	Target animal with passive shedding of residual vaccine virus due to bait consumption
T_NV_	Target animal with no passive shedding of residual vaccine, but at risk for vaccine-induced rabies (not yet seroconverted)
T_R_	Target animal rabid due to vaccine-induced reversion
T_S_	Target animal with no passive shedding of residual vaccine and not at risk for developing vaccine induced rabies
NT_V_	Non-target animal with passive shedding of residual vaccine virus due to bait consumption
NT_NV_	Non-target animal with no passive shedding of residual vaccine, but at risk for vaccine-induced rabies (not yet seroconverted)
NT_R_	Non-target animal rabid due to vaccine-induced rabies
NT_S_	Non-target animal with no passive shedding of residual vaccine and not at risk for developing vaccine induced rabies
H_MC_	Immuno-competent/immuno-compromised human inoculated due to mucosal contact with vaccine
H_TC_	Immuno-competent/immuno-compromised human inoculated due to transdermal contact with vaccine
H_L_	Immuno-competent/immuno-compromised human with mucosal membrane or fresh wound licked by vaccinated animal
H_B_	Immuno-competent/immuno-compromised human bitten by vaccinated animal
H_SB_	Immuno-competent/immuno-compromised human severely bitten by vaccinated animal with cranial or peritoneal inoculation
H_RB_	Immuno-competent/immuno-compromised human bitten by animal rabid due to vaccine induced rabies
H_SAE_	Immuno-competent/immuno-compromised human with severe adverse event
H_C,MC_	Immuno-competent/immuno-compromised human that receives medical care after mucosal contact with vaccine
H_C,TC_	Immuno-competent/immuno-compromised human that receives medical care after transdermal contact with vaccine
H_C,L_	Immuno-competent/immuno-compromised human that receives medical care after being licked by recently vaccinated animal
H_C,B_	Immuno-competent/immuno-compromised human that receives medical care after being bitten by recently vaccinated animal
H_C,SB_	Immuno-competent/immuno-compromised human that receives medical care after being bit severely by recently vaccinated animal
H_C,RB_	Immuno-competent/immuno-compromised human that receives medical care after being bit by animal rabid due to vaccine induced rabies
H_C,SAE_	Immuno-competent/immuno-compromised human that receives medical care after experiencing an vaccine-related SAE
H_R_	Immuno-competent/immuno-compromised human in end-stage safe state (recovered from SAE or no longer at risk for SAE)
H_D_	Immuno-competent/immuno-compromised human that dies due to vaccine-related SAE

After a bait (denoted B, in the model) is distributed, it can follow one–or more if bait is punctured and not swallowed–of these pathways: (1) punctured or eaten by target animal (denoted T_v_); (2) punctured or eaten by non-target animal (NT_v_); (3) not swallowed by an animal, but recovered by the distributor (B_r_) and either reused (B) or discarded (B_NV_); (4) left in the environment (B_v_) either intact, or after being punctured (e.g. not swallowed) by an animal. Baits left in the environment have some risk period during which the vaccine is viable and could result in inoculation following mucosal or transdermal contact with humans. It is assumed that even punctured baits carry some risk. After this risk period passes, the vaccine is considered non-infectious (B_NV_).

We assumed that no animal consumes more than one bait and that all animals that puncture the bait receive an adequate dose of the vaccine to seroconvert. Upon perforation of the bait sachet, the vaccine is released into the oral cavity where viable vaccine virus can be found for a limited period of time after bait consumption^32,33^. At this stage, a transdermal bite or a lick on an open wound or the mouth from the vaccinated animal could place a human at risk for exposure to the live vaccine. This risk period is allowed to vary in the model, but typically lasts not longer than 24 hours for the vaccines assessed in this study (Supplemental Table 3), after which the animal may no longer expose someone via contact with the oral cavity (T_nv_ and NT_nv_)^32^. Once the vaccine is cleared from the oral cavity and the animal has seroconverted, contact with the vaccinated animal no longer poses a risk for SAEs to people (T_S_ and NT_S_). For some live vaccines, such as SAD-B19, there is low probability that the vaccine virus escapes clearance by the immune system and has sufficient residual pathogenicity that it may induce rabies in the vaccinated animals (T_R_ and NT_R_). Death rate of rabid animals and vaccine-induced rabies transmission events to humans were included in the model.

Human compartments, shown in red, are stratified by immune-competent and immune-compromised individuals (denoted by the double boxes). These compartments reflect the six different inoculation pathways via which an SAE may occur. It is assumed that initially susceptible humans (H_S_), if exposed, are only exposed through one pathway per exposed person. These pathways are: (1) direct vaccine contact with the nose, eyes or mouth through physical handling of the vaccine (i.e. mucosal contact) (H_MC_); (2) direct vaccine contact with a cut or skin lesion resulting in transdermal innoculation (H_TC_); (3) contamination of a mucosal membrane or fresh wound with residual vaccine from a recently vaccinated animal (here, from the lick of an animal with residual vaccine-virus contaminated saliva) (H_L_); (4) bite from recently vaccinated animal with residual live vaccine in the oral cavity resulting in transdermal inoculation (H_B_); (5) severe bite from an animal with residual vaccine-virus in the oral cavity resulting in inoculation into the cranial or peritoneal cavity (H_SB_); or (6) bite from an animal that developed vaccine-induced infection (here, rabies) (H_RB_).

The exposed human may either seek care or not (H_C,__X,_ *where X denotes exposure route*), where the probability of seeking care varies based on exposure route. For this rabies model, we assumed those who seek care do so immediately, and that those who seek care will be treated with post exposure prophylaxis (PEP) and/or rabies immunoglobulin (RIG) according to WHO recommendations. Accordingly, we assumed PEP and RIG are readily available in a country and is 100% effective except in cases of severe bite penetrating the cranial or peritoneal cavity, where it is only 30% effective. Exposed humans that do not seek care are at risk for developing an SAE (H_SAE_). The probability of the SAE differs according to the type of exposure and immune state of the affected person. Values are derived from laboratory studies of adverse reactions in immuno-competent and -compromised mice (Supplemental Tables 4 and 5). Exposed humans that do not seek care may still not develop an SAE, due to the complex host-virus interactions that must be completed for successful infection (H_R_). To accommodate other less lethal live vaccines, the model allows for a person to recover post SAE, at a rate consistent with the vaccine-strain considered.

For this rabies virus-based model, it is assumed all persons with an SAE will succumb to rabies virus infection (H_D_). However, the model also allows for the development of an SAE post care, or for the person to seek care post experiencing an SAE (H_C,SAE_). In this way, the model allows for different vaccines to be simulated. The model also permits simulations with different target animal populations, including both domestic (dog) and wildlife targets. By taking into account characteristics of the human population and the magnitude of the planned campaign, as described below, it is applicable to a variety of settings.

### Model parameters

Estimates for parameter values describing the rate/probability of a human leaving one compartment and entering the other have been taken from the various literature sources and are displayed in Supplemental Table 2.

The key parameters that differ between SAD-B19 and SPBN GASGAS campaigns include those related to probability of SAE post exposure. Values for number of hours live vaccine remains in the animal oral cavity and probability of SAE in immuno-compromised and immune-competent individuals are drawn from laboratory inoculation experiments with immune compromised and immune competent mice, the results of which are shown in Supplemental Tables 3–5^32^.

Six animals have been confirmed rabid post SAD-B19 consumption in the field across a total of 277 million SAD-B19 baits distributed over the past 35 years^11,18,34^. We multiplied this documented rate of reversion to rabies by a factor of 2 to account for the fact that not all rabid animals bite people, a factor of 10 to account for under-reporting of rabid wildlife, and another factor of 10 to account for the number of baits that never reached a target. The third generation oral rabies virus vaccine SPBN GASGAS has retained the immunogenic properties of its parental strain SAD-B19 but has a greatly improved safety profile in comparison to first and second generation oral rabies virus vaccines. The risk of reversion to virulence of SPBN GASGAS is negligible based on its specific genetic design in the form of targeted multiple genetic modifications^2,35^, its greater genetic stability^35^, and evidence from experimental safety studies^2,32,36^. Furthermore, mutations in the parental SAD-B19 genome of vaccine viruses isolated from vaccine induced rabies cases were genetically very similar to potent SAD vaccines that have undergone a history of *in vitro* selection^11^, and very likely were not the result of the selection of a more virulent genotype, as corroborated in experimental studies^18^.

The key parameters that differ between dog and fox campaigns are the uptake rate of the target and non-target animals, the bite rate of the target and non-target animals, and the lick rate of the target and non-target animals. Human behavior characteristics as contact rate with vaccine baits left in the environment and health care seeking behavior following exposure to the vaccine via any of the six possible routes are included based on field studies^37,38^ and household surveys^39^, respectively.

### Simulations and initial conditions

A continuous-time stochastic Markov chain was simulated using the standard Gillespie algorithm^40^. In R, 1,000 iterations were run using a 400,000 bait campaign conducted over a 60 day period. The time period was allowed to extend 21 days after campaign ended, or until no more movement between compartments was possible (i.e. all baits, vaccinated animals, and exposed humans had reached a terminal compartment). Solutions from every iteration were recorded, and the mean, 95% confidence intervals, and ranges are reported. The size of the simulated campaign (400,000) was based on the number of baits needed to vaccinate 20% of dogs in a hypothetical setting consistent with the human population of the average canine rabies endemic country (excluding China and India) and using a human-to-dog ratio of 11:1 (estimated to be 22,000,000 people)^41,42^. Presumably the remaining dogs would be unvaccinated or vaccinated via direct injection. We assumed a closed population of dogs and humans. The size of the simulated campaigns for wildlife campaigns was kept at 400,000 for the purposes of comparability between campaign types,

Proportion of immunocompromised individuals were calculated from literature reporting percentage of the population living with blood cancers^43^, HIV with CD4 count < 200^44–46^, and severe primary immunodeficiency disorder^47^. Since HIV rate largely drove the percent of immunocompromised persons, we used estimated prevalence of persons living with CD4 count < 200 in Europe and South Africa for the low and high values of the sensitivity range, respectively^44–46^. We assumed none of the population had been previously vaccinated against rabies and thus everyone was susceptible.

### Sensitivity Analysis

Sensitivity analysis was conducted to explore the effect of parameter uncertainty on the outcomes of interest. A range of possible values was given for each parameter where there was uncertainty in the estimate (Supplemental Table 2). Ranges for the sensitivity analysis were selected (in order of priority) on the basis of: (1) range of values provided in the literature; (2) 95% confidence interval on a well-defined proportion in the literature; (3) ± 50% of the estimate for fairly well-defined values without a definite proportion; (4) a factor of 10 above and below the estimate for not well-defined values.

Latin hypercube sampling was used to draw 1,000 samples from the total range of possible values of each parameter using the ‘lhs’ package in R^48^. Latin hypercube sampling is a method of sensitivity analysis that allows for more randomness than systematic sampling, while ensuring uniform coverage of the entire range of uncertainty for all of the parameters which contain uncertainty. For those parameters where the expected value fell to the lower end of the possible range of values, values were sampled from between log transformed ranges and then back transformed.

After running the Markov chain with the 1,000 different parameter combinations, the partial rank correlation coefficient (prcc) was determined for each of the parameters using the sensitivity package in R^49^. The prcc reports the influence of each parameter on the outcome of interest; here, the number of exposed persons, the number of persons seeking care, and the number of people with an SAE. Bootstrapping with 1,000 iterations was used to obtain 95% confidence intervals for the correlation coefficient. A Bonferroni adjustment was applied for significance estimations, adjusting for the number of parameters varied in the sensitivity analysis.

### Disclaimer

The findings and conclusions in this report are those of the authors and do not necessarily represent the official position of CDC.

## Results

**Figure 2:**
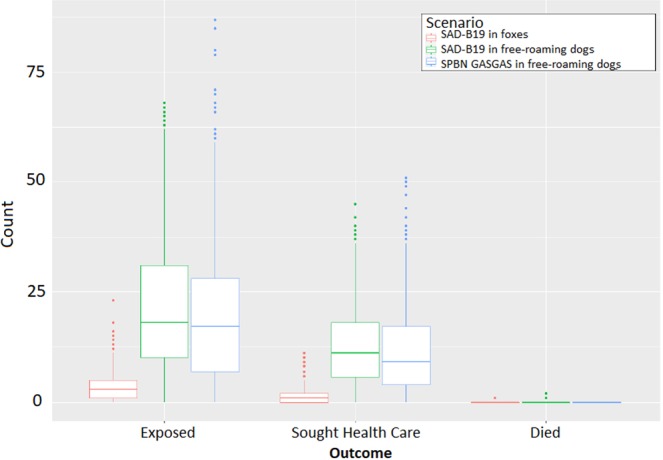
Boxplots of outcomes for each of the 1,000 Markov chain runs under each ORV scenario using the standard model. Centered lines show the median value for each of the 1,000 runs, upper and lower edges of the box indicate the interquartile range (IQR), and the lines indicate the 95% confidence intervals. Dots represent outliers.

We conducted 1,000 simulations of each of the three ORV campaign scenarios (400,000 baits per campaign). Distribution of the counts of each of three outcomes of interest (exposures, health care visits, and deaths) obtained per simulation were heavily right skewed (Fig. 2). Analysis of the change in median and rate values per additional simulation show that 1,000 simulations was sufficient to reach stability (Supplemental Fig. 1).

### SAD-B19 with foxes as target population

First, we simulated a campaign using SAD-B19 vaccines with foxes as the target population, and other wildlife and domestic species (e.g. dogs) as the non-target population. Over the 1,000 simulated campaigns each with 400,000 baits, a mean of 79,983 (20.0%) baits were simulated as left unconsumed in the environment, 100,003 baits (25.0%) were consumed by foxes (the target species), and 220,003 baits (55.0%) were consumed by a non-target animal. A mean of 3.4 human exposures (95% CI: 0, 12; Range: 0, 23) per campaign were simulated (Table 2). The majority of these simulated exposures were from direct physical contact with the bait in the environment, either on the mouth, eyes or nose (i.e. mucosal inoculation) (mean: 1.0; 95% CI: 0, 5; Range: 0, 11) or on a cut or skin lesion (i.e. transdermal inoculation) (mean: 1.1; 95% CI: 0, 6; Range: 0, 11). A mean of 1.6 exposure-associated health care visits (95% CI: 0, 6; Range: 0, 11), and a mean of 0.007 deaths (Range: 0, 1) per campaign were simulated. When summing over all 1,000 simulations, the model simulated only seven deaths, corresponding to a very low death rate of 0.18 (95% CI: 0.08, 0.36) per 10 million baits distributed. Of these seven simulated deaths, five (71.4%) were attributable to either mucosal or transdermal inoculation after direct physical contact with the vaccine in the environment, and the remaining two (28.6%) were attributable to a bite from an animal that had developed vaccine-induced rabies. In all simulations for this hypothetical population, per every ten million baits distributed, 5.3 (95% CI: 4.6, 6.1) foxes and 13.1 (95% CI: 12.0, 14.3) non-target animals were simulated to experience vaccine-induced rabies.

**Table 2:** Simulation results for human exposures and severe adverse events from three different campaign situations.

Vaccine use	Human Exposures Mean (95% CI); Median (Med); Range (R); Sensitivity Analysis Range (SR)							Exposure-prompted visits to health care facilities Mean (95% CI); Med; R; SI	Exposure-associated deaths	
	Total	Mucosal contact	Transdermal contact	Transdermal bite	Severe bite	Lick	Bite from rabid animal	Total	Total Count	Rate
									Mean (95% CI); Med; R; SI	Rate per 10 million baits (95% CI)
**SAD B19** ***(Foxes as targets)***	**3**.**31 (0,12)**	**1**.**05 (0,3)**	**1**.**13 (0,3)**	**1**.**11 (0,5)**	**0 (0,0)**	**0**.**004 (0,1)**	**0**.**017 (0,1)**	**1**.**59 (0,6)**	**0**.**007 (0,1)**	**0**.**18** (0.08, 0.36)
	Med: 3	Med: 1	Med: 1	Med: 1	Med: 0	Med: 0	Med: 0	Med: 1	Med: 0	
	R: 0–23	R: 0–5	R: 0–8	R: 0–8	R: 0	R: 0–1	R: 0–2	R: 0–11	R: 0–1	
	SR: 0–333	SR: 0–91	SR: 0–147	SR: 0–162	SR: 0–1	SR: 0–8	SR: 0–2	SR: 0–155	SR: 0–19	
**SAD-B19** ***(Dogs as targets)***	**21**.**50 (2,54)**	**1**.**01 (0,5)**	**1**.**09 (0,6)**	**17**.**65 (1,51)**	**0 (0,0)**	**0**.**06 (0,1)**	**1**.**68 (0,7)**	**12**.**54 (1,33)**	**0**.**13 (0,1)**	**3**.**35** (2.83, 3.98)
	Med: 18	Med: 0	Med: 0	Med: 15	Med: 0	Med: 0	Med: 1	Med: 18	Med: 0	
	R: 0–68	R: 0–12	R: 0–12	R: 0–64	R: 0–1	R: 0–2	R: 0–13	R: 0–45	R: 0–2	
	SR: 1–219	SR: 0–191	SR: 0–165	SR: 0–142	SR: 0–1	SR: 0–6	SR: 0–46	SR: 0–127	SR: 0–61	
**GASGAS** ***(Dogs as targets)***	**19**.**37 (2,54**.**2)**	**0**.**45 (0,3)**	**0**.**43 (0,3)**	**18**.**42 (1,54)**	**0 (0,0)**	**0**.**06 (0,1)**	**0 (0,0)**	**11**.**36 (1,32)**	**0 (0,0)**	**0** (0, 0.12)
	Med: 17	Med: 0	Med: 0	Med: 16	Med: 0	Med: 0	Med: 0	Med: 9	Med: 0	
	R: 2–87	R: 0–8	R: 0–5	R: 0–87	R: 0	R: 0–2	R: 0	R: 0–51	R: 0	
	SR: 1–1,128	SR: 0–144	SR: 0–128	SR: 1–1,046	SR: 0	SR: 0–9	SR: 0	SR: 0–775	SR: 0–1	

### SAD-B19 with dogs as target population

Next, we simulated a campaign of using SAD-B19 vaccines with free-roaming dogs as the target population. A mean of 21.5 exposures (95% CI: 2, 54; Range: 0, 68) per campaign were simulated (Table 1) for SAD-B19 campaigns in dog populations. The majority of these simulated exposures were bites from dogs who had recently consumed the vaccine (mean: 17.7; 95% CI: 1, 49), followed by bites from dogs who had developed vaccine-induced rabies (mean: 1.7; 95% CI: 0, 7). A mean of 12.5 exposure-associated health care visits (95% CI: 1, 33; Range: 0, 68), and 0.13 deaths (95% CI: 0, 1; Range: 0, 2) per campaign were simulated. When summing over all 1,000 simulations, a human death rate of 3.4 (95% CI: 2.8, 4.0) per 10 million baits was simulated. This rate was 18.6 higher than the rate modeled for SAD-B19 in fox populations. Of these simulated deaths, 91.0% were attributable to bites from dogs that developed vaccine-induced rabies. The remaining 9% were from bites from dogs who had recently consumed the vaccine (3.0%), and mucosal or transdermal contact with the vaccine during direct handling of the vaccine in the environment (6.0%). In all simulations, 40.2 (95% CI: 38.3, 42.2) dogs per every 10 million baits distributed experienced vaccine-induced rabies.

### SPBN GASGAS in dog populations

No human deaths occurred when simulating the SPBN GASGAS campaign in a dog target population. Similar to the SAD-B19 vaccine for use in dog target populations, a mean of 19.4 exposures (95% CI: 2, 54; Range: 0, 87) per campaign were simulated (Table 2). The majority of these simulated exposures were bites from dogs who had recently consumed the vaccine (mean: 18.4; 95% CI: 1, 51). A mean of 11.4 exposure-associated health care visits (95% CI: 1, 32; Range: 0, 51) were simulated. Given that we assumed negligible reversion to virulence based on the genetic composition of SPBN GASGAS as well as evidence from safety studies or field trials, in no simulations were animals simulated to experience vaccine-induced rabies.

### Sensitivity analysis

**Figure 3:**
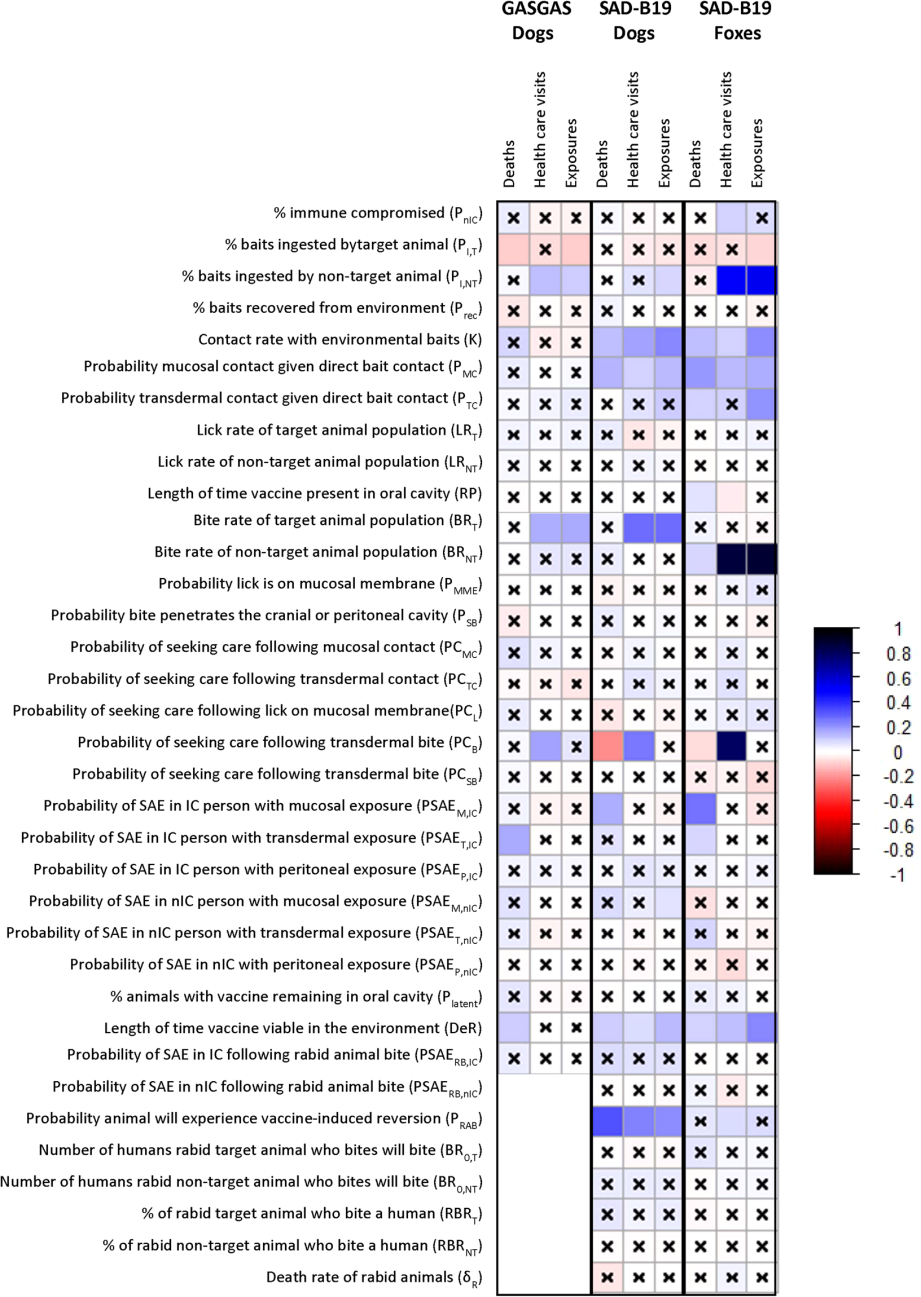
Correlation plot showing sensitivity of number of simulated exposures, health care visits, and deaths to parameters in the model. Color indicates the value of the partial rank correlation coefficient. Squares without an x are significant at the 95% confidence level, after Bonferroni adjustment for the number of parameters considered.

We used Latin hypercube sampling and partial rank correlation tests to examine the effect of parameter uncertainty across the possible range of each parameter. Figure 3 indicates the partial rank correlation coefficient (prcc) and Bonferroni-adjusted 95% confidence interval produced during the sensitivity analysis, and Table 2 shows the ranges for the values produced in the sensitivity analysis. For SAD-B19 in fox populations, the most important exposure route was contact with baits that had been left in the environment. Numbers of SAEs simulated was most sensitive to changes in human contact rate with bait in the environment (prcc = 0.13), probability of direct mucosal (prcc = 0.20) or transdermal (prcc = 0.09) contact given contact with bait, rate of decay of bait in the environment (prcc = 0.10), bite rate of the non-target animals (e.g. dogs) (prcc = 0.09), and probability of an SAE given direct contact either resulting in mucosal (prcc = 0.26) or transdermal (prcc = 0.08) innoculation. Numbers of exposures and health care visits (HCV) simulated was most sensitive to human contact rate with bait in the environment (Exposures: prcc = 0.22; HCV: prcc = 0.10), probability of mucosal contact given contact with bait (Exposures: prcc = 0.16; HCV: prcc = 0.14), and rate of decay of bait in the environment (Exposures: prcc = 0.23; HCV: prcc = 0.13). Higher consumption of baits by foxes reduced exposures (Exposures: prcc = −0.08), while higher consumption of baits by non-target animals increased exposures and health care visits (Exposures: prcc = 0.53; HCV: prcc = 0.51). Numbers of health care visits was also sensitive to the probability of seeking healthcare after a bite (prcc = 0.80), and higher health care seeking behavior helped reduce the number of SAEs simulated (prcc = −0.04).

For SAD-B19 in dog populations, the most important exposure route was bites from rabid dogs that had developed vaccine-induced rabies. Numbers of deaths simulated was most sensitive to the probability a dog will undergo a vaccine-induced rabies infection (prcc = 0.33), and the probability that a person will seek care following a bite (prcc = −0.21). Changes in parameters involved in the direct contact pathway, including contact rate with bait left in environment (prcc = 0.13), probability of mucosal contact given bait contact (prcc = 0.14), and probability of an SAE following direct bait contact with a mucosal membrane (prcc = 0.15) also affected the number of deaths simulated. Changes in bite rate of the target animal significantly affected the number of simulated exposures (prcc = 0.27) and health care visits (prcc = 0.27), as did changes in the probability of vaccine induced reversion (Exposures: prcc = 0.22; HCV: prcc = 0.24). Direct contact with a bait was also an important parameter (Exposures: prcc = 0.22, HCV: prcc = 0.18). The number of health care visits was also sensitive to the probability of seeking care following an animal bite (prcc = 0.25), which also reduced deaths (prcc = −0.21).

For SPBN GASGAS in dog populations, the most important exposure route was bites from dogs recently consuming the bait. The number of exposures simulated was most sensitive to the dog bite rate (prcc = 0.16). The number of health care visits was most sensitive to the dog bite rate (prcc = 0.16) and the probability of seeking care following a dog bite (prcc = 0.18). The only death simulated in the sensitivity analysis was due to direct contact with the bait, so the number of deaths simulated in sensitivity analysis were sensitive to parameters relating to contact (uptake of bait by target species which dictates how many baits are left in the environment, contact rate with bait in environment, probability of severe adverse event given transdermal contact, and decay rate of bait in the environment).

## Discussion

We developed and applied a novel method to compare the risk of various oral vaccines that can be used to aid planning of vaccination campaigns for wild or domestic animal populations. Application of the model may be helpful for conducting risk assessments prior to the distribution of oral vaccines in the environment or to explore downstream effects of changes to the vaccine, bait, or distribution method of existing oral vaccination programs. We compared a first generation oral rabies vaccine, SAD-B19 as an example vaccine, to a heavily attenuated, third generation vaccine, SPBN GASGAS, as well as compared application of these vaccines for the purposes of rabies control in wildlife populations compared to rabies control in free-roaming dog populations. Consistent with international concern over the use of first generation oral rabies vaccines in dog populations, our model predicts that using SAD-B19 to control rabies in free-roaming dogs exhibits an 18.6 times higher risk of a SAE in humans compared to the use of the same vaccine for campaigns targeted at fox populations. This finding is not unexpected and reflects the dynamic roles contact rates and exposure risk play. Mass distribution in the environment (such as aerial distribution in wildlife vaccination programs) may increase the risk of contact with a bait in the environment However, the risk of vaccine exposure and subsequent SAEs are lower for these type of contacts. In comparison, individual bait hand-out distribution (as used for dog vaccination programs) reduces the number of baits available for contact in the environment but vaccinates animals with more frequent and closer contact with humans.

Safer vaccines with less residual pathogenicity can mitigate risks associated with higher risk distribution models. Unlike the SAD-B19 vaccine, the model for dog vaccination with SPBN GASGAS did not project a substantial rate of SAEs. In the sensitivity analysis, only one death per 400 million baits (or 0.025 per 10 million baits) was simulated and only under extreme parameter selections that would be unlikely to occur in a real-world setting. We expect the exposures and health care visits for dog vaccination programs with SAD-B19 and SPBN GASGAS to be similar, or slightly lower in SPBN GASGAS, as the exposure route of bites from dogs that had experienced vaccine-induced rabies, which was a relatively rare event for SAD-B19, was null for SPBN GASGAS. This similarity of exposures and health care visits was observed in our model. Even though SAEs from pathways other than a bite from a rabid dog was low for SAD-B19, it did occur at a rate of about 30 deaths per billion baits distributed. This was higher than that for SPBN GASGAS, where zero deaths were predicted.

We find that bait consumption and dog bite rate are consistently among the most sensitive parameters in dog bait hand-out campaigns, while bait consumption, human contact rate, and decay rate of vaccine in the environment are consistently among the most sensitive parameters in wildlife campaigns. This is not unexpected as Babzadeh *et al*. reports that 95.2% of human bites are from dogs, while only 1% are from wildlife (and 0.1% from foxes)^50^. This is important not only for producing more accurate individual country model simulations, but also for exploring and quantifying the impact of new bait technologies and distribution methods to reduce SAEs. Distribution methods in wildlife that increase uptake by the target species while reducing non-target uptake will not only increase vaccination efficiency, but also have a measurable impact on reducing human exposures and subsequent SAEs. For instance, the design of baits for vaccination programs targeting fox populations should be highly palatable for foxes, but not to dogs. Furthermore, campaign planners should prioritize public education to not touch baits it if they are found in the environment. Considerable effort is spent by planners of wildlife vaccination campaigns regarding the placement and baiting density of vaccination zones. Targeted education and exposure mitigation efforts can be designed as well. In the European fox campaigns for instance, the planners informed dog owners to keep their dogs inside for several days during the campaign to reduce the risk of non-target bait uptake and mitigate the risk of human exposures. These unique preventive measures could be modeled in future, country-specific, modeling exercises to estimate the beneficial impact of specific bait-avoidance interventions.

A conservative estimation approach was used when there was uncertainty regarding parameters to not underestimate the potential risk of SAEs in the model. For instance, with a simulated death rate of 0.18 (95% CI: 0.08, 0.36) per ten million baits of SAD-B19 distributed to fox populations, SAEs from SAD-B19 in wildlife are an extremely rare, but possible event. Over 277 million baits of SAD-B19 have been used to control rabies within the fox populations in 11 European countries over a 31-year period with no reported human deaths^34^. Model validation without any recorded cases poses a challenge and thus was not attempted here; however, it can be noted that both the model and field experience agree that the risk posed to humans is exceedingly low. While human rabies is a rare event and has a relatively high detectability compared to other rare pathogens, cases go unreported and mis-diagnosed. In the United States, which operates one of the world’s most advanced public health rabies control programs, approximately half of the human rabies deaths are thought to go unrecognized^51^.

For the purposes of being able to compare a simulated campaign of SAD-B19 in wildlife to simulated campaigns of SAD-B19 and SPBN GASGAS in dog populations, we selected our parameters to be representative of a general country where canine rabies is endemic, and not to reflect the unique parameters of the 11 European countries where SAD-B19 was used, particularly in terms of health care seeking behavior, proportion of patients immunocompromised, and proportion of baits left in the environment. The latter, which is significantly positively associated with SAE in sensitivity analysis, is expected to be especially important in overestimating vaccine-associated deaths as our model simulated that 20% of baits were left in the environment, while some studies from Europe have shown that the proportion remaining is often less than 20%, and could drop to nearly zero within seven days after distribution^52–54^. While point-estimate validation of the fox model was not appropriate here, the finding that SAD-B19 was associated with a nearly 19-fold increased risk when used for dog campaigns compared to foxes is consistent with international guidance discouraging use of first generation live attenuated rabies vaccines for dog vaccination^10^.

The value describing the probability of developing an SAE given exposure to the oral rabies vaccine is estimated from a limited number of mice safety studies, resulting in large 95% confidence intervals. We attempted to overcome this and other uncertainties with sensitivity analysis, in which we show that the value for the probability of a SAE given an exposure is less important than the values of parameters relating to the exposure itself (i.e. contact rate with baits, dog bite rate, bait consumption). This finding suggests that even vaccines which are less safe in vaccine safety studies may be safe when used in a safe context. The reverse could also be true in that even vaccines which appear safe in safety studies may cause risk if used in a manner which promotes exposures. This highlights the deficiency in current ORV safety assessment criteria and the utility of incorporating a model like this into the decision on whether or not a vaccine formulation should be used. However, if any new safety study data is made publicly available, the risk assessment should be revised.

Our model makes several assumptions which may also cause variation in the estimated risk. First, the assumption that each animal consumes only one bait may overestimate the risk of human SAE during dog campaigns, as it maximizes the number of dogs that can expose a human to the vaccine. Multiple doses do not increase the risk of vaccine-induced rabies, since overdose studies (high dose, repeated dose) were safe in target species. We also assume that RIG and PEP are readily available, would be sought immediately by a large proportion of the exposed population, and are universally recommended, which may underestimate rates of SAEs, especially in low income countries where medical resources may be difficult to access^55^. However, we argue that ORV should only be conducted after the community is notified of the campaign and has available to them appropriate post-contact health-care assessments. Finally, for simplicity, the model does not account for rabies caused by bites from animals that were secondary-transmission rabies cases – in other words, from those animals that became rabid due to a bite from an animal who had developed vaccine-induced rabies. Inclusion of this pathway could increase the SAE rate among humans, but is thought to be an extremely rare event. Studies inoculating juvenile foxes with a SAD-B19 isolate from a vaccine induced case yielded only seroconversion in the foxes and no secondary rabies cases^18^.

This model has several strengths and could be helpful in performing risk assessments for a variety of oral animal vaccines when used in conjunction with appropriate safety and efficacy testing in laboratory and controlled field experiments. It is flexible enough to be applied to other oral rabies vaccines that utilize different formulations, such as adenovirus or vaccinia virus, where the SAE may not always be death, but some other less severe (but more common) condition^56,57^. For instance, humans have come into contact with environmentally distributed recombinant oral rabies vaccines utilizing vaccinia virus for vaccine delivery and have developed skin lesions at the location of contact^58,59^. Similarly, contact with vaccines using adenovirus for vaccine delivery could induce respiratory symptoms^57^. In these scenarios, the model allows for the SAE to develop prior to seeking care. The model can also be used to simulate human safety of oral vaccination of both domestic and wildlife animals. This has the potential to significantly aid demonstration of safety for a variety of animal vaccines, such as those for bovine tuberculosis, brucellosis, plague, and anthrax, while also quickly deterring researchers from spending undue resources field testing vaccines that will not be appropriately safe. Through sensitivity analysis, the model permits comparison of vaccination campaigns, even when there is uncertainty surrounding the parameters.

In conclusion, this is the first attempt to create a standardized methodology to compare simulated risks to humans posed by the use of oral vaccines in animal populations. The model has several applications beyond those explored here. For instance, future study could use the model to explore the human safety of recombinant oral rabies vaccines that utilize adenovirus or vaccinia virus, to compare other (non-rabies) oral vaccines in both domestic and wild animals, or to personalize the model to a specific country context. The model is also flexible enough to be adapted to other modified-live, virus vaccines that have a potential for environmental introduction. Most readily comparable include oral vaccination of animals against other zoonotic disease. However, examination of shedding of poliovirus post oral polio vaccination could also be investigated by making small modifications such as the concept of animal uptake to human uptake, and animal shedding of virus via saliva to fecal shedding of the virus. Finally, the results of our simulations on the oral rabies vaccine support international concern that first generation oral rabies vaccines, which pose little risk when used in wildlife populations, may cause undue risk to humans when used in dog populations. However, the model highlights the safety of a third generation SPBN GASGAS rabies vaccine, even when used to control rabies in dog populations.

## Supplementary information

Supplemental Info

## Data Availability

All data needed for simulation of campaigns is provided in the Supplemental Files. Any person wishing to utilize the R shiny tool we have developed to improve ease of running our simulations is welcome to contact J.R.H. (jrhead6@gmail.com) or RW (euk5@cdc.gov).
