# Supplementary material for: Environmental distribution of certain modified live-virus vaccines with a high safety profile presents a low-risk, high-reward to control zoonotic diseases
Source: Sci Rep. 2019 May 1;9:6783. doi: 10.1038/s41598-019-42714-9 (PMC6494895; doi:10.1038/s41598-019-42714-9)
Supplement: Supplementary file 1 — Supplemental Info [file 41598_2019_42714_MOESM1_ESM.pdf]

Supplementary Information for

Environmental distribution of certain modified live-virus vaccines with a high safety profile presents a low-risk, high-reward to control zoonotic diseases

Authors: Jennifer R. Head, Ad Vos, Jesse Blanton, Thomas Müller, Richard Chipman, Emily G. Pieracci, Julie Cleaton, Ryan Wallace

Jennifer R. Head
Email: jrhead6@gmail.com

This PDF file includes:

Supplementary text
Figs. S1
Tables S1 to S5
References for SI reference citations

Supplementary Information Text

Methods.

Supplementary tables in this document provide the mathematical equations and associated parameter values, governing the probability of movement of a bait, animal, or human between the compartments shown in the diagram in Figure 1 of the text. They contain information on each parameter value used in the mathematical equation as well as their justification for use, based on literature or laboratory results. Results of laboratory studies examining shedding of the rabies virus in dog saliva as well as the challenge studies of mice with both SAD-B19 and SPBN GASGAS oral rabies vaccines are included here.

Results.

A figure showing that rates of exposures, health care visits and deaths stabilizes as more and more campaigns are simulated is provided as justification that the model was run a sufficient number of simulations.

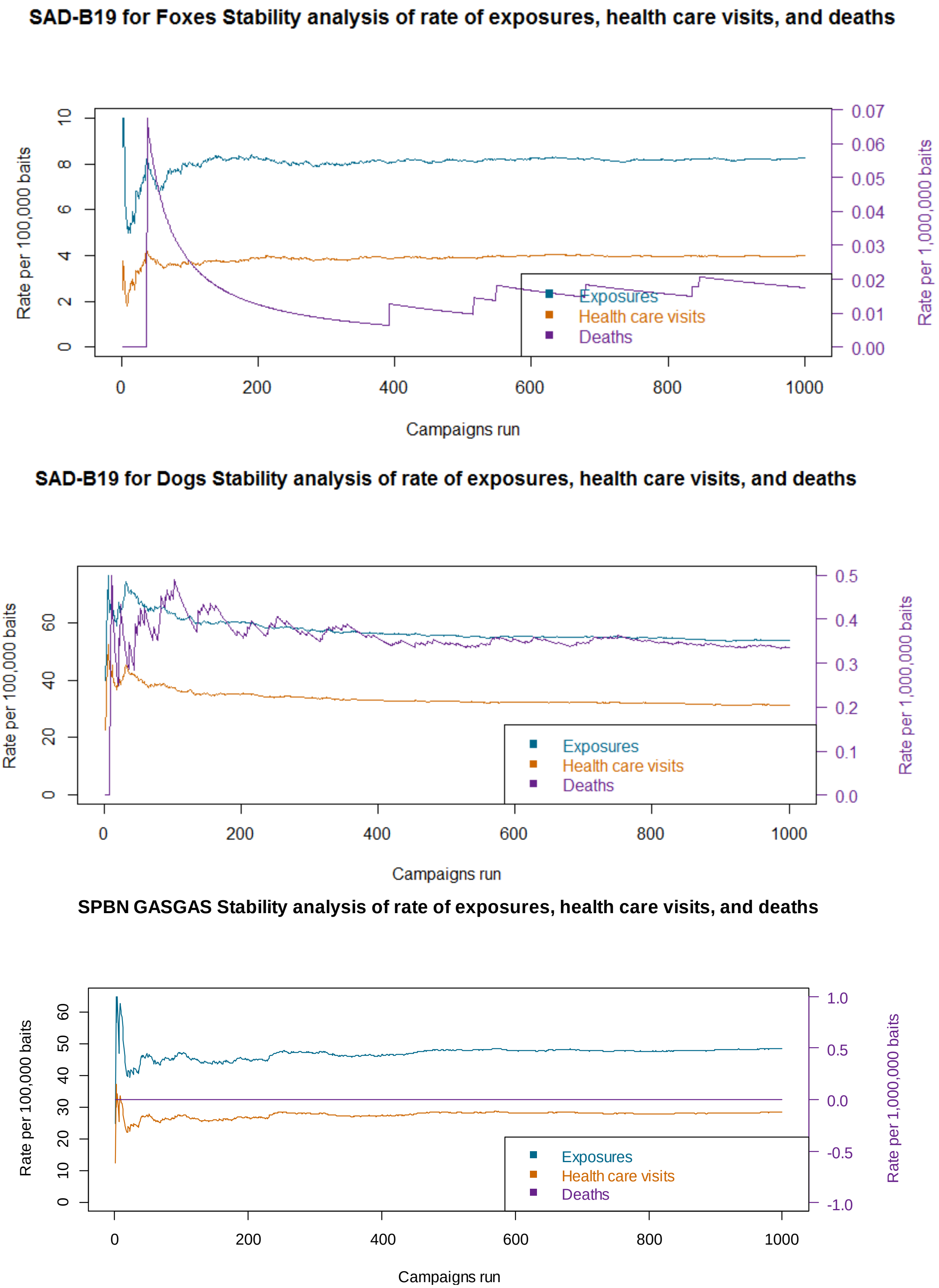

Fig. S1. Stability analysis demonstrating changes in rate of outcomes measured per number of simulations run

Table S1. Mathematical equations describing rate of movement between each model compartment

#	Rate Equation	Description	#	Rate Equation	Description
1	$DiR \bullet P_{rec} \bullet (1-(P_{SWALLOW} \bullet P_{I,T} + P_{I,NT}))$, if B > 0, 0 else	recovery of baits in enrvironment	48	$(1-PC_{L}) \bullet P_{latent} \bullet PSAE_{M,nIC} \bullet H_{L,nIC}$	nIC with mucosal lick gets SAE
2	$P_{reuse} \bullet B_{r}$	reuse of recovered bait	49	$(1-PC_{B}) \bullet P_{latent} \bullet PSAE_{T,nIC} \bullet H_{TB,nIC}$	nIC with transdermal bite gets SAE
3	$(1/DeR) \bullet (1-P_{reuse}) \bullet B_{R\|time,\ t = DeR}$,	destruction of recovered bait	50	$(1 - P_{SB}) \bullet P_{latent} \bullet PSAE_{P,nIC} \bullet H_{SB,nIC}$	nIC with severe bite gets SAE
	if $B_{R} > 0$ & time, $t \geq DeR$, 0 else		51	$(1 - PC_{B}) \bullet PSAE_{RB,IC} \bullet H_{RB,nIC}$	nIC gets SAE after rabid animal bite
4	$DiR \bullet (1-P_{rec}) \bullet (1-(P_{SWALLOW} \bullet P_{I,T} + P_{I,NT}))$, if B > 0, 0<br>else	bait left in environment	52	$(1 - PC_{MC} - PSAE_{M,IC}) \bullet H_{MC,IC}$	IC with mucosal contact avoids SAE w/o care
5	$(1/DeR) \bullet B_{V\|time,\ t = DeR}$,	decay of left bait	53	$(1-PC_{TC} - PSAE_{T,IC}) \bullet H_{TC,IC}$	IC with transdermal contact avoids SAE w/o care
	if $B_{V} > 0$ & time, $t \geq DeR$, 0 else, 0 else		54	$(1-PC_{L}-(P_{latent} \bullet PSAE_{M,IC})) \bullet H_{L,IC}$	IC with mucosal lick avoids SAE w/o care
6	$DiR \bullet P_{I,T}$, if B > 0, 0 else	target ingests bait	55	$(1-PC_{B}-(P_{latent} \bullet PSAE_{T,IC})) \bullet H_{TB,IC}$	IC with transdermal bite avoids SAE w/o care
7	$(1/RP) \bullet (1-P_{RAB}) \bullet T_{V\|time,\ t = RP}$,	target stops shedding in oral cavity	56	$(1-PC_{SB}-(P_{latent} \bullet PSAE_{P,IC})) \bullet H_{SB,IC}$	IC with severe bite avoids SAE w/o care
	if $T_{V} > 0$ & time, $t \geq RP$, 0 else		57	$(1-PC_{B}-PSAE_{RB,nIC}) \bullet H_{RB,nIC}$	IC bitten by rabid dog avoids SAE without care
8	$P_{RAB} \bullet T_{NV}$	target becomes rabid	58	$(1 - PC_{MC} - PSAE_{M,nIC}) \bullet H_{MC,nIC}$	nIC with mucosal contact avoids SAE w/o care
9	$(1-P_{RAB}) \bullet T_{NV}$	target becomes seroconverts	59	$(1-PC_{TC} - PSAE_{T,nIC}) \bullet H_{TC,nIC}$	nIC with transdermal contact avoids SAE w/o care
10	$\delta_{R} \bullet T_{R}$	death of rabid target animal	60	$(1-PC_{L}-(P_{latent} \bullet PSAE_{Mn,IC})) \bullet H_{L,nIC}$	nIC with mucosal lick avoids SAE w/o care
11	$DiR \bullet P_{I,NT}$, if B > 0, 0 else	non-target ingests bait	50	$(1 - P_{SB}) \bullet P_{latent} \bullet PSAE_{P,nIC} \bullet H_{SB,nIC}$	nIC with severe bite gets SAE
12	$(1/RP) \bullet (1-P_{RAB}) \bullet NT_{V\|time,\ t = RP}$,	non-target stops shedding in oral cavity	62	$(1-PC_{SB}-(P_{latent} \bullet PSAE_{P,IC})) \bullet H_{SB,IC}$	nIC with severe bite avoids SAE w/o care
	if $NT_{V} > 0$ & time, $t \geq RP$, 0 else		63	$(1-PC_{B}-PSAE_{RB,IC}) \bullet H_{RB,IC}$	nIC bitten by rabid dog avoids SAE without care
13	$P_{RAB} \bullet NT_{NV}$	non-target becomes rabid	64	$\alpha \bullet PSAE_{M,IC} \bullet H_{C,MC,IC}$	IC with mucosal contact gets SAE after seeking care
14	$(1-P_{RAB}) \bullet NT_{NV}$	non-target seroconverts	65	$\alpha \bullet PSAE_{T,IC} \bullet H_{C,TC,IC}$	IC with transdermal contact gets SAE after seeking care
15	$\delta_{R} \bullet NT_{R}$	death of rabid non-target animal	66	$\alpha \bullet P_{latent} \bullet PSAE_{M,IC} \bullet H_{C,L,IC}$	IC with mucosal lick gets SAE after seeking care
16	$(1-P_{nIC}) \bullet K \bullet P_{MC} \bullet B_{V}$	IC exposed mucosally (direct contact)	67	$\alpha \bullet P_{latent} \bullet PSAE_{T,IC} \bullet H_{C,TB,IC}$	IC with transdermal bite gets SAE after seeking care
17	$(1-P_{nIC}) \bullet K \bullet P_{TC} \bullet B_{V}$	IC exposed transdermally (direct contact)	68	$\alpha_{p} \bullet P_{latent} \bullet PSAE_{P,IC} \bullet H_{C,SB,IC}$	IC with severe bite gets SAE after seeking care
18	$(1-P_{nIC}) \bullet LR_{T} \bullet P_{MME} \bullet T_{V} + (1-P_{nIC}) \bullet LR_{NT} \bullet P_{MME} \bullet NT_{V}$	IC exposed via lick on mucosal membranes	69	$[(P_{SB} \bullet \alpha_{p} \bullet PSAE_{P,IC}) + ((1-P_{SB}) \bullet \alpha \bullet PSAE_{RB,IC}))] \bullet H_{C,RB,IC}$	IC with rabid bite gets SAE after seeking care
19	$(1-P_{nIC}) \bullet BR_{T} \bullet (1-P_{SB}) \bullet T_{V} + (1-P_{nIC}) \bullet BR_{NT} \bullet (1-P_{SB}) \bullet NT_{V}$	IC exposed via transdermal bite	70	$\alpha \bullet PSAE_{M,nIC} \bullet H_{C,MC,nIC}$	nIC with mucosal contact gets SAE after seeking care
20	$(1-P_{nIC}) \bullet BR_{T} \bullet P_{SB} \bullet T_{V} + (1-P_{nIC}) \bullet BR_{NT} \bullet P_{SB} \bullet NT_{V}$	IC exposed via severe bite	71	$\alpha \bullet PSAE_{T,IC} \bullet H_{C,TC,IC}$	nIC with transdermal contact gets SAE after care
21	$(1-P_{nIC}) \bullet (RBR_{T} \bullet BR_{0,T} \bullet T_{D} + RBR_{NT} \bullet BR_{0,NT} \bullet NT_{D})$	IC bitten by vaccine-induced rabid animal	72	$\alpha \bullet P_{latent} \bullet PSAE_{M,IC} \bullet H_{C,L,IC}$	nIC with mucosal lick gets SAE after care
22	$P_{nIC} \bullet K \bullet P_{MC} \bullet B_{V}$	nIC exposed mucosally (direct contact)	73	$\alpha \bullet P_{latent} \bullet PSAE_{T,IC} \bullet H_{C,TB,IC}$	nIC with transdermal bite gets SAE after care
23	$P_{nIC} \bullet K \bullet P_{TC} \bullet B_{V}$	nIC exposed transdermally (direct contact)	74	$\alpha_{p} \bullet P_{latent} \bullet PSAE_{P,IC} \bullet H_{C,SB,IC}$	nIC with severe bite gets SAE after seeking care
24	$P_{nIC} \bullet LR_{T} \bullet P_{MME} \bullet T_{V} + P_{nIC} \bullet LR_{NT} \bullet P_{MME} \bullet NT_{V}$	nIC exposed via lick on mucosal membranes	75	$[(P_{SB} \bullet \alpha_{p} \bullet PSAE_{P,IC}) + ((1-P_{SB}) \bullet \alpha \bullet PSAE_{RB,IC}))] \bullet H_{C,RB,IC}$	nIC with rabid bite gets SAE after seeking care
25	$P_{nIC} \bullet BR_{T} \bullet (1-P_{SB}) \bullet T_{V} + P_{nIC} \bullet BR_{NT} \bullet (1-P_{SB}) \bullet NT_{V}$	nIC exposed via transdermal bite	76	$(1- \alpha \bullet PSAE_{M,IC}) \bullet H_{C,MC,IC}$	IC with mucosal contact seeks care and avoids SAE
26	$P_{nIC} \bullet BR_{T} \bullet P_{SB} \bullet T_{V} + P_{nIC} \bullet BR_{NT} \bullet P_{SB} \bullet NT_{V}$	nIC exposed via severe bite	77	$(1- \alpha \bullet PSAE_{T,IC}) \bullet H_{C,TC,IC}$	IC with transdermal contact seeks care and avoids SAE
27	$P_{nIC} \bullet (RBR_{T} \bullet BR_{0,T} \bullet T_{D} + RBR_{NT} \bullet BR_{0,NT} \bullet NT_{D})$	nIC bitten by vaccine-induced rabid animal	78	$(1- \alpha \bullet PSAE_{M,IC}) \bullet H_{C,L,IC}$	IC with mucosal lick seeks care and avoids SAE
28	$PC_{MC} \bullet H_{MC,IC}$	IC exposed mucosally gets care	79	$(1- \alpha \bullet PSAE_{T,IC}) \bullet H_{C,TB,IC}$	IC with transdermal bite seeks care and avoids SAE
29	$PC_{TC} \bullet H_{TC,IC}$	IC exposed transdermally gets care	80	$(1- \alpha_{p} \bullet PSAE_{P,IC}) \bullet H_{C,SB,IC}$	IC with severe bite seeks care and avoids SAE
30	$PC_{L} \bullet H_{L,IC}$	IC with mucosal lick gets care	81	$[(1-(P_{SB} \bullet \alpha_{p} \bullet PSAE_{P,IC} +(1-P_{SB}) \bullet \alpha \bullet PSAE_{RB,IC}))] \bullet H_{C,RB,IC}$	IC with rabid bite seeks care and avoids SAE
31	$PC_{B} \bullet H_{TB,IC}$	IC with transdermal bite gets care	82	$(1- \alpha \bullet PSAE_{M,nIC}) \bullet H_{C,MC,nIC}$	nIC with mucosal contact seeks care and avoids SAE
32	$PC_{SB} \bullet H_{SB,IC}$	IC with severe bite gets care	83	$(1- \alpha \bullet PSAE_{T,nIC}) \bullet H_{C,TC,nIC}$	nIC with transdermal contact seeks care and avoids SAE
33	$PC_{B} \bullet H_{RB,IC}$	IC with rabid animal bite gets care	84	$(1- \alpha \bullet PSAE_{M,nIC}) \bullet H_{C,L,nIC}$	nIC with mucosal lick seeks care and avoids SAE
34	$PC_{MC} \bullet H_{MC,nIC}$	nIC exposed mucosally gets care	85	$(1- \alpha \bullet PSAE_{T,nIC}) \bullet H_{C,TB,nIC}$	nIC with transdermal bite seeks care and avoids SAE
35	$PC_{TC} \bullet H_{TC,nIC}$	nIC exposed transdermally gets care	86	$(1- \alpha_{p} \bullet PSAE_{P,nIC}) \bullet H_{C,SB,nIC}$	nIC with severe bite seeks care and avoids SAE
36	$PC_{L} \bullet H_{L,nIC}$	nIC with mucosal lick gets care	87	$[(1-(P_{SB} \bullet \alpha_{p} \bullet PSAE_{P,nIC}+(1-P_{SB}) \bullet \alpha \bullet PSAE_{RB,nIC}))] \bullet H_{C,RB,nIC}$	nIC with rabid bite seeks care and avoids SAE
37	$PC_{B} \bullet H_{TB,nIC}$	nIC with transdermal bite gets care	88	$PC_{SAE} \bullet H_{SAE,IC}$	IC with SAE seeks care
38	$PC_{SB} \bullet H_{SB,nIC}$	nIC with severe bite gets care	89	$PC_{SAE} \bullet H_{SAE,nIC}$	nIC with SAE seeks care

39	$PC_{B} \bullet H_{RB,nIC}$	nIC with rabid animal bite gets care	90	$\Upsilon_{SAE,IC} \bullet H_{SAE,IC}$	IC with SAE recovers w/o care
40	$(1-PC_{MC}) \bullet PSAE_{M,IC} \bullet H_{MC,IC}$	IC with mucosal contact gets SAE	91	$\Upsilon_{SAE,nIC} \bullet H_{SAE,nIC}$	nIC with SAE recovers w/o care
41	$(1-PC_{TC}) \bullet PSAE_{T,IC} \bullet H_{TC,IC}$	IC with transdermal contact gets SAE	92	$\Upsilon_{C,IC} \bullet H_{C,SAE,IC}$	IC with SAE recovers after care
42	$(1-PC_{L}) \bullet P_{latent} \bullet PSAE_{M,IC} \bullet H_{L,IC}$	IC with mucosal lick gets SAE	93	$\Upsilon_{C,nIC} \bullet H_{C,SAE,nIC}$	nIC with SAE recovers after care
43	$(1-PC_{B}) \bullet P_{latent} \bullet PSAE_{T,IC} \bullet H_{TB,IC}$	IC with transdermal bite gets SAE	94	$(1-\Upsilon_{SAE,IC}) \bullet H_{SAE,IC}$	IC with SAE dies w/o additional care
44	$(1-P_{SB}) \bullet P_{latent} \bullet PSAE_{P,IC} \bullet H_{SB,IC}$	IC with severe bite gets SAE	95	$(1-\Upsilon_{SAE,nIC}) \bullet H_{SAE,nIC}$	nIC with SAE dies w/o additional care
45	$(1-PC_{B}) \bullet PSAE_{RB,IC} \bullet H_{RB,IC}$	IC gets SAE after rabid animal bite	96	$(1-\Upsilon_{C,IE}) \bullet H_{C,SAE,IC}$	IC with SAE dies after care
46	$(1-PC_{MC}) \bullet PSAE_{M,nIC} \bullet H_{MC,nIC}$	nIC with mucosal contact gets SAE	97	$(1-\Upsilon_{C,nIE}) \bullet H_{C,SAE,nIC}$	nIC with SAE dies after care
47	$(1-PC_{TC}) \bullet PSAE_{T,nIC} \bullet H_{TC,nIC}$	nIC with transdermal contact gets SAE			

IC = immune competent

nIC = not immune competent (immune compromised)

Table S2. Parameter values for each parameter in Table SA1, along with justification for values used in the oral rabies vaccination comparison

Parameter	Description	Value (Range)			Source/Justification
		SAD B19 in foxes	SAD B19 in dogs	SPBN GASGAS in dogs	
P_{IC}	Proportion of immuno-compromised individuals	4.814E-3 (4.094E-3, 2.728E-2)	4.814E-3 (4.094E-3, 2.728E-2)	4.814E-3 (4.094E-3, 2.728E-2)	Based on the number of persons living with HIV [1] and depressed CD4 count [2,3], primary immune deficiency disorder [4], or blood cancers [5]; Sensitivity analysis considers HIV rate in South Africa and Europe
P_{vax}	Proportion of people vaccinated against disease	0 (fixed)	0 (fixed)	0 (fixed)	Most conservative estimate; Varies geographically
H_S0	Total human population	4,400,000 (fixed)	4,400,000 (fixed)	4,400,000 (fixed)	Based on a global average human to dog ratio of 11:1 [6]; Varies geographically
B_0	Total amount of bait	400,000 (fixed)	400,000 (fixed)	400,000 (fixed)	20% of total dog population are “inaccessible” dogs
DiR	Daily distribution rate of baits	6,667 (fixed)	6,667 (fixed	6,667 (fixed)	400,000 baits over 60 day campaign
P_{rec}	Proportion of non-ingested baits recovered from environment	0 (fixed)	0.263 (0.10 – 0.51)	0.263 (0.10 – 0.51)	DOG: Campaign results from Haiti [7]; Sensitivity analysis is 95% confidence interval around the proportion
P_{reuse}	Proportion of baits reused after recovery	N/A	0.50 (fixed)	0.50 (fixed)	Expert opinion
DeR	Number of days vaccine is viable in environment	10.0 (3, 30)	10.0 (3, 30)	10.0 (3, 30)	Laboratory field tests [8]
$P_{I,T}$	Proportion of baits ingested (punctured or swallowed) by target animal	0.20 (0.10, 0.30)	0.931 (0.89, 0.96)	0.931 (0.89, 0.96)	Results from previous campaigns [7,9-16]. Sensitivity analysis is 95% confidence interval around the proportion
$P_{SWALLOW}$	Among those baits ingested, proportion of fully swallowed	1 (fixed)	1 (fixed)	1 (fixed)	Held to isolate effect of animal consumption in this model
$P_{I,NT}$	Proportion of baits ingested by non-target animal	0.55 (0.40, 0.70)	3.436E-3 (2.0E-4, 2.2E-2)	3.436E-3 (2.0E-4, 2.2E-2)	Results from previous campaigns [7,9-16]. Sensitivity analysis is 95% confidence interval around the proportion
K	Daily human contact rate with the bait in the environment	4.847E-5 (2.891E-6, 7.358E-5)	1.107E-4 (6.6E-6, 1.68E-4)	1.107E-4 (6.6E-6, 1.68E-4)	DOG: Center and range drawn from data on calls about exposure from multistate ORV surveillance systems [17] and results of wildlife campaign in Ohio [18]. FOX: multiplied dog values by an adjustment factor to account for less dense applications [19-22]
P_{MC}	Probability the contact event results in mucosal inoculation	0.0714 (0.013, 0.31)	0.0714 (0.013, 0.31)	0.0714 (0.013, 0.31)	Results of exposure types from wildlife campaign in Ohio [18]; Sensitivity analysis is 95% confidence interval around proportion.
P_{TC}	Probability the contact event results in transdermal inoculation	0.071 (0.013, 0.31)	0.071 (0.013, 0.31)	0.071 (0.013, 0.31)	Results of exposure types from wildlife campaign in Ohio [18]; Sensitivity analysis is 95% confidence interval around proportion.
LR_T	Daily probability of being licked by an animal from the target population	5.480E-12 (2.736E-12, 8.208E-12)	4.110E-4 (2.055E-4, 6.164E-4)	4.110E-4 (2.055E-4, 6.164E-4)	FOX: Based on bite rate and rare event justification; Sensitivity analysis (±50% of estimate).

					DOG: Based on expert opinion from prevalence of dog ownership [23] and bite rate in Haiti [24]; Sensitivity analysis (±50% of estimate).
LR_{NT}	Daily probability of being licked by an animal from the non-target population	3.726E-5 (1.863E-5, 5.589E-5)	4.110E-6 (1.027E-6, 9.247E-6)	4.110E-6 (1.027E-6, 9.247E-6)	FOX: Based on weighted average of lick rate for dogs and wild animals; Sensitivity analysis (±50% of estimate).<br>DOG: Assumed 1% of target population; Sensitivity analysis (±50% of estimate).
RP	Days vaccine stays in oral cavity	0.5 (0.167, 1)	0.5 (0.167, 1)	0.5 (0.167, 1)	Shedding studies (Supplemental Table 2) [25]
P_{MME}	Probability of mucous membrane or wound exposure to saliva	6.667E-4 (6.667E-6, 6.667E-3)	6.667E-4 (6.667E-6, 6.667E-3)	6.667E-4 (6.667E-6, 6.667E-3)	Considered rare event
BR_{T}	Daily bite rate of target population	8.219E-8 (4.110E-8, 1.233E-7)	8.219E-5 (4.110E-5, 1.233E-4)	8.219E-5 (4.110E-5, 1.233E-4)	DOG: Household survey in Haiti [24]; Sensitivity analysis (±50% of estimate).<br>FOX: Adjustment from dog bite rate based on percent of rabid bites attributable to foxes [26]; Sensitivity analysis (±50% of estimate).
BR_{NT}	Daily bite rate of non-target population	8.151E-06<br>(4.075E-06, 1.223E-05)	9.863E-6 (2.466E-6, 2.219E-5)	9.863E-6 (2.466E-6, 2.219E-5)	FOX: Based on weighted average of lick rate for dogs and wild animals [26]; Sensitivity analysis (±50% of estimate).<br>DOG: Based on bite data from Los Angeles, CA [27]; Sensitivity analysis (±50% of estimate).
P_{SB}	Proportion of animal bites with cranial or peritoneal inoculation	5.60E-5 (2.80E-5, 1.68E-4)	5.60E-5 (2.80E-5, 1.68E-4)	5.60E-5 (2.80E-5, 1.68E-4)	Based on data from Los Angeles, CA regarding percentages of bites that resulted in hospitalization and to the face [27] and data from Paris on face bites that break the bone [28]; Sensitivity analysis (±50% of estimate).
P_{RAB}	Proportion of animals that develop vaccine induced rabies	2.360E-6 (2.360E-7, 2.360E-5)	4.332E-6 (4.332E-7, 4.332E-5)	0 (fixed)	FOX: Results of ORV campagins in foxes [13]<br>DOG: Adjustment from 6/277 million to account for underreporting; Sensitivity analysis (factor of 10 above and below estimate).
δ_{R}	Death rate of rabid animal	7.14E-2 (3.5E-2, 1.07)	7.14E-2 (3.5E-2, 1.07)	N/A	Death rate of rabid animals [29]; Sensitivity analysis (±50% of estimate).
RBR_{T}	Percent of rabid target animals that bite another human	3.653E-6	0.5 (0.25, 0.75)	0.5 (0.25, 0.75)	Expert opinion; Sensitivity analysis (±50% of estimate).
RBR_{NT}	Percent of rabid non-target animals that bite another human	4.5E-2 (2.0E-2, 7.5E-2)	3.653E-6 (3.653E-7, 3.653E-5)	3.653E-6 (3.653E-7, 3.653E-5)	Expert opinion; Sensitivity analysis (factor of 10 above and below estimate).
$BR_{0,T}$	Daily bite rate of rabid target animals (among those that bite)	7.14E-2 (3.5E-2, 1.07)	1.429E-1 (7.14E-2, 2.143E-1)	1.429E-1 (7.14E-2, 2.143E-1)	Expert opinion; Sensitivity analysis (±50% of estimate).
$BR_{0,NT}$	Daily bite rate of rabid non-target animals (among those that bite)	7.793E-2 (3.896E-2, 1.169E-1)	7.14E-2 (3.5E-2, 1.07)	7.14E-2 (3.5E-2, 1.07)	Expert opinion; Sensitivity analysis (±50% of estimate).
$PSAE_{M,IC}$	Probability of a severe adverse event if mucous membranes are exposed to vaccine in an immune competent person	7.4E-3 (3.15E-3, 5.5E-1)	7.4E-3 (3.15E-3, 5.5E-1)	9.0E-5 (9.0E-7, 9.0E-3)	Mouse challenge studies (SAD-B19: Supplemental Table 3; SPBN GASGAS: Supplemental Table 4) [30]
$PSAE_{M,nIC}$	Probability of a severe adverse event if mucous membranes are exposed to vaccine in an immune compromised person	4.84E-2 (2.1E-3, 1.7E-1)	4.84E-2 (2.1E-3, 1.7E-1)	3.78E-2 (1.1E-2, 3.78E-1)	Mouse challenge studies (SAD-B19: Supplemental Table 3; SPBN GASGAS: Supplemental Table 4) [30]

$PSAE_{T,IC}$	Probability of a severe adverse event with transdermal exposure to vaccine in an immune competent person	2.9E-3 (1.0E-5, 5.0E-2)	2.9E-3 (1.0E-5, 5.0E-2)	9.0E-5 (9.0E-7, 9.0E-3)	Mouse challenge studies (SAD-B19: Supplemental Table 3; SPBN GASGAS: Supplemental Table 4) [30]
$PSAE_{T,nIC}$	Probability of a severe adverse event with transdermal exposure to vaccine in an immune compromised person	9.98E-2 (6.15E-2, 9.2E-1)	9.98E-2 (6.15E-2, 9.2E-1)	1.42E-2 (1.42E-3, 1.42E-1)	Mouse challenge studies (SAD-B19: Supplemental Table 3; SPBN GASGAS: Supplemental Table 4) [30]
$PSAE_{P,IC}$	Probability of a severe adverse event with peritoneal or cranial exposure to vaccine in an immune competent person	9.99E-3 (7.51E-3, 9.5E-1)	9.99E-3 (7.51E-3, 9.5E-1)	9.0E-5 (9.0E-7, 9.0E-3)	Mouse challenge studies (SAD-B19: Supplemental Table 3; SPBN GASGAS: Supplemental Table 4) [30]
$PSAE_{P,nIC}$	Probability of a severe adverse event with peritoneal or cranial exposure to vaccine in an immune compromised person	1E-1 (8.32E-2, 1.0)	1E-1 (8.32E-2, 1.0)	7.79E-2 (7.79E-1, 3.66E-2)	Mouse challenge studies (SAD-B19: Supplemental Table 3; SPBN GASGAS: Supplemental Table 4) [30]
$PSAE_{RB,IC}$	Probability of a severe adverse event with following a bite from a rabid animal in an immune competent person	0.18 (0.09, 0.27)	0.18 (0.09, 0.27)	0.18 (0.09, 0.27)	Report [31]; Sensitivity analysis (±50% of estimate).
$PSAE_{RB,nIC}$	Probability of a severe adverse event with following a bite from a rabid animal in an immune compromised person	0.36 (0.18, 0.54)	0.36 (0.18, 0.54)	0.36 (0.18, 0.54)	Twice that for immune competent individuals (expert opinion).
P_{latent}	Probability animal had latent immune response	0.20 (0.10- 0.30)	0.20 (0.10- 0.30)	0.20 (0.10- 0.30)	Shedding studies (Supplemental Table 2) [25]
PC_{MC}	Probability an exposed person sought care after mucosal contact with vaccine	0.43 (0.05, 0.64)	0.43 (0.05, 0.64)	0.43 (0.05, 0.64)	Household survey on health-care seeking behavior in Haiti [32]; Sensitivity analysis based on before and after values
TC_{TC}	Probability an exposed person sought care after transdermal contact with vaccine	0.43 (0.05, 0.64)	0.43 (0.05, 0.64)	0.43 (0.05, 0.64)	Household survey on health-care seeking behavior in Haiti [32]; Sensitivity analysis based on before and after values
PC_{L}	Probability an exposed person sought care after licked on a mucosal membrane or fresh wound	0.44 (0.11, 0.80)	0.44 (0.11, 0.80)	0.44 (0.11, 0.80)	Household survey on health-care seeking behavior in Haiti [32]; Sensitivity analysis based on before and after values
PC_{B}	Probability an exposed person sought care after bite	0.60 (0.44, 0.99)	0.60 (0.44, 0.99)	0.60 (0.44, 0.99)	Household survey on health-care seeking behavior in Haiti [32]; Sensitivity analysis based on before and after values
PC_{SB}	Probability an exposed person sought care after severe bite resulting in cranial or peritoneal inoculation	0.90 (0.44, 0.99)	0.90 (0.44, 0.99)	0.90 (0.44, 0.99)	Household survey on health-care seeking behavior in Haiti [32]; Sensitivity analysis based on before and after values
PC_{SAE}	Probability a person with an SAE seeks care	0	0	0	For rabies only: assumed a person with an SAE dies
α	Probability of SAE after care is given, for a non cranial or peritoneal innoculation	0	0	0	For rabies only: assumed PEP is 100% effective if administed

α_P	Probability of SAE after care is given, for a cranial or peritoneal innoculation	0.70 (0.60, 0.80)	0.70 (0.60, 0.80)	0.70 (0.60, 0.80)	Expert opinion; sensitivity analysis ± 10%
$\Upsilon_{SAE,IC/nIC}$	Probability of recovery in persons with an SAE who don't seek care	0	0	0	For rabies only: assumed a person with an SAE dies
$\Upsilon_{SAE,IC/nIC}$	Probability of recovery in persons with an SAE after seeking care	0	0	0	For rabies only: assumed a person with an SAE dies

Table S3. Experimental data on shedding (saliva) studies with SPBN GASGAS [25]. All samples examined were pre-screened with PCR, and only PCR-positive samples were examined by RTCIT (hr – hours after vaccine administration).

Hours after exposure	Number of animals examined	Number of animals with rabies virus detected in saliva
**1**	24	4
**2**	104	13
**4**	105	21
**24**	168	0
**48**	163	0
**72**	147	0
**120**	32	0
**168**	62	0
**240**	35	0
**336**	42	0

Table S4. **Laboratory results of mice safety studies with SAD-B19 and associated model parameter choices**

Type of exposure to vaccine:	# mice exposure	# deaths	% of mice who died	Lower confidence Interval*	Lower confidence Interval*	Model lower bound	Model central bound	Model upper bound
**Immune competent mice:**								
mucosal membrane	20	11	0.55	0.32	0.754	0.00315	0.00754	0.55
transdermal	20	1	0.05	0.00	0.249	0.00001	0.00249	0.05
peritoneal or cranial	30	29	0.97	0.75	0.999	0.00751	0.00999	0.95
**Immune compromised or not yet immune competent (suckling) mice:**								
mucosal membrane	12	2	0.17	0.02	0.484	0.0021	0.0484	0.17
transdermal	12	11	0.92	0.62	0.998	0.0615	0.0998	0.92
peritoneal or cranial	20	20	1.00	0.83	1	0.0832	0.1	1

*Determined using fisher's exact test

Table S5. Results of mice safety studies with SPBN GASGAS and associated model parameter choices

Type of exposure to vaccine:	# mice exposed	# deaths	% of mice who died	Lower confidence Interval*	Lower confidence Interval*	Model lower bound	Model central bound	Model upper bound
**Immune competent mice:**								
mucosal membrane	20	0	0.00	0.00	0.168	0	0.00009	0.009
transdermal	87	0	0.00	0.00	0.041	0	0.00009	0.009
peritoneal or cranial	418	0	0.00	0.00	0.009	0	0.00009	0.009
**Immune compromised or not yet immune competent (suckling) mice:**								
mucosal membrane	44	10	0.23	0.11	0.378	0.011	0.0378	0.378
transdermal	24	0	0.00	0.00	0.142	0	0.0142	0.142
peritoneal or cranial	24	14	0.58	0.37	0.779	0.0366	0.0779	0.779

*Determined using fisher's exact test

References

1 Fettig, J., Swaminathan, M., Murrill, C. S. & Kaplan, J. E. Global Epidemiology of HIV. *Infectious disease clinics of North America* **28**, 323-337, doi:10.1016/j.idc.2014.05.001 (2014).

2 Kranzer, K., Lawn, S. D., Johnson, L. F., Bekker, L.-G. & Wood, R. Community viral load and CD4 count distribution among people living with HIV in a South African township: implications for treatment as prevention. *Journal of acquired immune deficiency syndromes (1999)* **63**, 498-505, doi:10.1097/QAI.0b013e318293ae48 (2013).

3 Malaza, A., Mossong, J., Bärnighausen, T., Viljoen, J. & Newell, M.-L. Population-Based CD4 Counts in a Rural Area in South Africa with High HIV Prevalence and High Antiretroviral Treatment Coverage. *PloS one* **8**, e70126, doi:10.1371/journal.pone.0070126 (2013).

4 Kobrynski, L., Powell, R. W. & Bowen, S. Prevalence and morbidity of primary immunodeficiency diseases, United States 2001-2007. *Journal of clinical immunology* **34**, 954-961, doi:10.1007/s10875-014-0102-8 (2014).

5 Leukemia and Lymphoma Society. *Facts and statistics*, <http://www.lls.org/facts-and-statistics/> (2018).

6 Wallace, R. M., Undurraga, E. A., Blanton, J. D., Cleaton, J. & Franka, R. Elimination of Dog-Mediated Human Rabies Deaths by 2030: Needs Assessment and Alternatives for Progress Based on Dog Vaccination. *Frontiers in veterinary science* **4**, 9, doi:10.3389/fvets.2017.00009 (2017).

7 Smith, T. G. *et al.* Evaluation of immune responses in dogs to oral rabies vaccine under field conditions. *Vaccine*, doi:https://doi.org/10.1016/j.vaccine.2017.09.096 (2017).

8 Vos, A. & Neubert, A. Thermo-stability of the oral rabies virus vaccines SAD B19 and SAD P5/88. *DTW. Deutsche tierarztliche Wochenschrift* **109**, 428-432 (2002).

9 Steelman, H. G., Henke, S. E. & Moore, G. M. Bait delivery for oral rabies vaccine to gray foxes. *Journal of wildlife diseases* **36**, 744-751, doi:10.7589/0090-3558-36.4.744 (2000).

10 Brochier, B. *et al. A field trial in Belgium to control fox rabies by oral immunisation*. Vol. 123 (1989).

11 Wandeler, A. I., Pfotenhauer, P. & Stocker, C. Über die Verwendung von Ködern zu biologischen Untersuchungen an Füchsen. *Revue suisse Zool* **82**, 335-348 (1975).

12 Paquot, A., Brochier, B., Thomas, I. & Pastoret, P. P. Campagnes de vaccination antirabique du renard roux (vulpes vulpes) menées en Belgique: mise en evidence de l'ingestion d'appats vaccinaux par le cerf rouge (Cervus elaphus), le chevreuil (Capreolus capreolus) et le sanglier (Sus scrofa). *Ann Med Vet* **132**, 697-702 (1988).

13 Müller, T. *et al.* Analysis of vaccine-virus-associated rabies cases in red foxes (Vulpes vulpes) after oral rabies vaccination campaigns in Germany and Austria. *Archives of virology* **154**, 1081-1091, doi:10.1007/s00705-009-0408-7 (2009).

14 Müller, T. *et al.* Testung eines neuen Köders für die orale Immunisierung des Rotfuchses (Vulpes vulpes) gegen Tollwut Berl. *Munch. Tierarztl. Wochenschr.* **106**, 41-46 (1993).

15 Dedek, J., Loepelmann, H., Loepelmann, F., Kühne, L. & Rex, M. Praxisbericht über den Nachweis von Oxytetracyclin in Knochenspänen von Wildtieren mittels Fluoreszenzmikroskopie. *Monatsh. Veterinarmed.* **46**, 8-10 (1991).

16 Stöhr, K., Stöhr, P. & Müller, T. Orale Fuchsimpfung gegen Tollwut - Ergebnisse und Erfahrungen aus den ostdeutschen Bundesländern. *Tierarztl. Umsch.* **4**, 203-211 (1994).

17 Roess, A. A. *et al.* National surveillance for human and pet contact with oral rabies vaccine baits, 2001-2009. *Journal of the American Veterinary Medical Association* **240**, 163-168, doi:10.2460/javma.240.2.163 (2012).
18 Kellog, F. *et al.* Human contacts with oral rabies vaccine baits distributed for wildlife rabies management - Ohio, 2012. *Morbidity and Mortality Weekly Report* **62**, 267-269 (2013).
19 Müller, T. F., Schröder, R., Wysocki, P., Mettenleiter, T. C. & Freuling, C. M. Spatio-temporal Use of Oral Rabies Vaccines in Fox Rabies Elimination Programmes in Europe. *PLOS Neglected Tropical Diseases* **9**, e0003953, doi:10.1371/journal.pntd.0003953 (2015).
20 Freuling, C. M. *et al.* Efficacy of the oral rabies virus vaccine strain SPBN GASGAS in foxes and raccoon dogs. *Vaccine,* doi:https://doi.org/10.1016/j.vaccine.2017.09.093 (2017).
21 Update on oral vaccination of foxes and raccoon dogs against rabies. *EFSA Journal* **13**, 4164, doi:doi:10.2903/j.efsa.2015.4164 (2015).
22 European Commission. The oral vaccination of foxes against rabies. *Report of the Scientific Committee on Animal Health and Animal Welfare* (2002).
23 Fielding, W. J., Gall, M., Green, D. & Eller, W. S. Care of Dogs and Attitudes of Dog Owners in Port-au-Prince, the Republic of Haiti. *Journal of Applied Animal Welfare Science* **15**, 236-253, doi:10.1080/10888705.2012.683760 (2012).
24 Schildecker, S. *et al.* Dog Ecology and Barriers to Canine Rabies Control in the Republic of Haiti, 2014-2015. *Transboundary and emerging diseases* **64**, 1433-1442, doi:10.1111/tbed.12531 (2017).
25 Vos, A. *et al.* An assessment of shedding with the oral rabies virus vaccine strain SPBN GASGAS in target and non-target species. *Vaccine* **36**, 811-817, doi:https://doi.org/10.1016/j.vaccine.2017.12.076 (2018).
26 Babazadeh, T. *et al.* Epidemiology of acute animal bite and the direct cost of rabies vaccination. *Journal of Acute Disease* **5**, 488-492, doi:https://doi.org/10.1016/j.joad.2016.08.019 (2016).
27 Lyu, C. *et al.* Burden of Bites by Dogs and Other Animals in Los Angeles County, California, 2009-2011. *Public Health Reports* **131**, 800-808, doi:10.1177/0033354916675148 (2016).
28 Touré, G., Angoulangouli, G. & Méningaud, J.-P. Epidemiology and classification of dog bite injuries to the face: A prospective study of 108 patients. *Journal of Plastic, Reconstructive & Aesthetic Surgery* **68**, 654-658, doi:https://doi.org/10.1016/j.bjps.2015.01.001 (2015).
29 Tepsumethanon, V. *et al.* Survival of Naturally Infected Rabid Dogs and Cats. *Clinical Infectious Diseases* **39**, 278-280, doi:10.1086/421556 (2004).
30 Faber, M. *et al.* A Single Amino Acid Change in Rabies Virus Glycoprotein Increases Virus Spread and Enhances Virus Pathogenicity. *Journal of Virology* **79**, 14141-14148, doi:10.1128/jvi.79.22.14141-14148.2005 (2005).
31 Babes, V. Traité de la rage. in: BN Fields, DM Knipe (Eds.) Virology. 2nd edn., (Raven Press, New York, NY, 1990).
32 Etheart, M. D. *et al.* Effect of counselling on health-care-seeking behaviours and rabies vaccination adherence after dog bites in Haiti, 2014–15: a retrospective follow-

up survey. *The Lancet Global Health* **5**, e1017-e1025, doi:10.1016/S2214-109X(17)30321-2.
